# Quality of life in neurotypical siblings of children with an autism spectrum disorder: Detecting children at risk

**DOI:** 10.1192/j.eurpsy.2021.1689

**Published:** 2021-08-13

**Authors:** E. Koukouriki

**Affiliations:** 1 Special Education Lab, Department Of Primary Education, University of Ioannina, Ioannina, Greece; 2 Centre For Educational And Counseling Services Of Trikala, Hellenic Ministry of Education and Religious Affairs, Trikala, Greece

**Keywords:** Quality of life, siblings, autism spectrum disorders

## Abstract

**Introduction:**

Quality of life (QOL) instruments can provide unique information on various aspects of children’s everyday functioning beyond clinical symptoms and clinical measures. The study of the QOL in the siblings of individuals with an Autism Spectrum Disorder (ASD-siblings) is a new direction in the field of ASD-research.

**Objectives:**

This study aimed to investigate the QOL in ASD-siblings in comparison with an age and sex matched group of siblings of neurotypical children.

**Methods:**

The sample included 233 neurotypical children (8-13 years old) of whom 118 comprised the observational group (ASD-siblings) and 115 comprised the comparison group. The Kidscreen-27 and a demographics questionnaire were administered to all participants.

**Results:**

The two groups differed significantly in all subscales and in the overall score of the Kidscreen-27, with the children in the observation group having a significantly worse quality of life (Mean±sd 111,62±12,43, p<0,001). Additionally, the largest difference in the averages of the subscales occurs in the subscale General Mood and Emotions, where the observation group had significantly lower scores (Mean±sd 23,23±3,69, p<0,001) than the comparison group (Mean±sd 31,27±2,74).

**Conclusions:**

QOL in children has been recognized as an important outcome indicator in detecting subgroups of children at risk within the general population, while ASD-siblings’ QOL is an important concept in the implementation of appropriate services for these children. This study revealed poorer QOL in ASD-siblings and highlighted the importance of assessing QOL in those siblings as well as the use of the Kidscreen-27 as a screening tool in order to detect children at risk of maladjustment.

**Disclosure:**

No significant relationships.

